# Detection of Mycotoxin Contamination in Foods Using Artificial Intelligence: A Review

**DOI:** 10.3390/foods13203339

**Published:** 2024-10-21

**Authors:** Ashish Aggarwal, Akanksha Mishra, Nazia Tabassum, Young-Mog Kim, Fazlurrahman Khan

**Affiliations:** 1 School of Bioengineering and Biosciences, Lovely Professional University, Phagwara 144001, Punjab, India; ashish.28916@lpu.co.in (A.A.); akanksha.42200033@lpu.in (A.M.); 2 Marine Integrated Biomedical Technology Center, The National Key Research Institutes in Universities, Pukyong National University, Busan 48513, Republic of Korea; nazia99@pukyong.ac.kr (N.T.); ymkim@pknu.ac.kr (Y.-M.K.); 3 Research Center for Marine Integrated Bionics Technology, Pukyong National University, Busan 48513, Republic of Korea; 4 Department of Food Science and Technology, Pukyong National University, Busan 48513, Republic of Korea; 5 Ocean and Fisheries Development International Cooperation Institute, Pukyong National University, Busan 48513, Republic of Korea; 6 International Graduate Program of Fisheries Science, Pukyong National University, Busan 48513, Republic of Korea

**Keywords:** mycotoxin detection, artificial intelligence, deep learning, machine learning, spectroscopy, chromatography, hyperspectral imaging, food safety, multi-mycotoxin detection

## Abstract

Mycotoxin contamination of foods is a major concern for food safety and public health worldwide. The contamination of agricultural commodities employed by humankind with mycotoxins (toxic secondary metabolites of fungi) is a major risk to the health of the human population. Common methods for mycotoxin detection include chromatographic separation, often combined with mass spectrometry (accurate but time-consuming to prepare the sample and requiring skilled technicians). Artificial intelligence (AI) has been introduced as a new technique for mycotoxin detection in food, providing high credibility and accuracy. This review article provides an overview of recent studies on the use of AI methods for the discovery of mycotoxins in food. The new approach demonstrated that a variety of AI technologies could be correlated. Deep learning models, machine learning algorithms, and neural networks were implemented to analyze elaborate datasets from different analytical platforms. In addition, this review focuses on the advancement of AI to work concomitantly with smart sensing technologies or other non-conventional techniques such as spectroscopy, biosensors, and imaging techniques for rapid and less damaging mycotoxin detection. We question the requirement for large and diverse datasets to train AI models, discuss the standardization of analytical methodologies, and discuss avenues for regulatory approval of AI-based approaches, among other top-of-mind issues in this domain. In addition, this research provides some interesting use cases and real commercial applications where AI has been able to outperform other traditional methods in terms of sensitivity, specificity, and time required. This review aims to provide insights for future directions in AI-enabled mycotoxin detection by incorporating the latest research results and stressing the necessity of multidisciplinary collaboration among food scientists, engineers, and computer scientists. Ultimately, the use of AI could revolutionize systems monitoring mycotoxins, improving food safety and safeguarding global public health.

## 1. Introduction

Mycotoxin contamination endangers the food supply and global public health. Most of these toxic secondary metabolites are produced by fungi such as *Aspergillus*, *Penicillium*, and *Fusarium* species and are found in a wide range of agricultural products such as cereals, nuts, spices, and dried fruits. Human and animal studies have shown that exposure to mycotoxins from the consumption of contaminated food is related to both acute detrimental effects (e.g., hepatotoxicity and nephrotoxicity) and chronic toxic outcomes (carcinogenicity and immunosuppression) [1,2,3].

Traditional methodologies of mycotoxin detection, such as chromatography with mass spectrometry (e.g., high-performance liquid chromatography (HPLC), gas chromatography–mass spectrometry (GC-MS), liquid chromatography–mass spectrometry (LC-MS), enzyme-linked immunosorbent assay (ELISA), etc.) are well known due to their good sensitivity and pattern recognition criteria; however, they are pricy, time-consuming processes derived from enzymes and require specifically trained personnel. These methods have complicated sample preparation steps that use extractions, purifications, and analyses that may suffer from variability and errors. Furthermore, this model necessitates fancy equipment and consumables that exceed the cost duty level in resource-limited settings, in addition to requiring regular monitoring in food production chains [4].

In recent years, the field of artificial intelligence (AI) has grown rapidly, becoming a viable contender to transform scientific research domains such as electroencephalographic (EEG) analysis [5], tumor detection [6], fungus identification [7], and biofilm detection and identification [8]. AI technologies expand the possibility of improving food safety by enabling advanced identification and tracking of mycotoxins in human foods [9,10,11]. Artificial intelligence refers to various computational methods and techniques that allow computers to evaluate and learn from data and find patterns to gain the capacity to make decisions nearly independently of humans. Machine learning (ML) techniques such as supervised learning, including methodologies such as support vector machines (SVM), random forest (RF), and neural networks (NNs), are starting to show promise in processing complicated datasets created by various analytical methods [12,13,14].

AI has garnered attention for its capacity to automatically extract intricate features from large data volumes, making it particularly suitable for image recognition, spectroscopic analysis, and the interpretation of sensor data for mycotoxin detection [15,16]. Advanced deep learning (DL) is a subset of AI algorithms inspired by the human brain’s structure and function [17]. DL models such as convolutional neural networks (CNNs), GoogLeNet, SqueezeNet, AlexNet, ResNet50, etc., have also shown promising results in processing complicated datasets. The use of these AI-driven techniques holds the promise of enhanced accuracy and sensitivity, as well as the possibility of quickly detecting and classifying mycotoxins in a variety of food matrices [18].

This review aims to offer a complete overview of current developments in artificial intelligence applications for the measurement of mycotoxin contamination in food products. It explores the integration of AI with diverse analytical platforms, including spectroscopy, biosensors, and imaging techniques, to enhance the efficiency, reliability, and speed of mycotoxin analysis. Furthermore, key challenges and limitations associated with AI-based methods, such as the need for robust datasets, standardization of protocols, and regulatory acceptance, are critically assessed.

The current review paper focuses on discussing the different aspects related to AI-based mycotoxin detection in detail, such as (1) the impact of AI techniques on the detection of mycotoxins in food using deep and machine learning, (2) a brief introduction to artificial intelligence and related techniques, (3) AI techniques for mycotoxin detection in food, (4) challenges associated with AI techniques for mycotoxin detection in food, and (5) several proposed future perspectives.

## 2. Impact of Artificial Intelligence Techniques in Detection of Mycotoxins in Food

**Figure 1:**
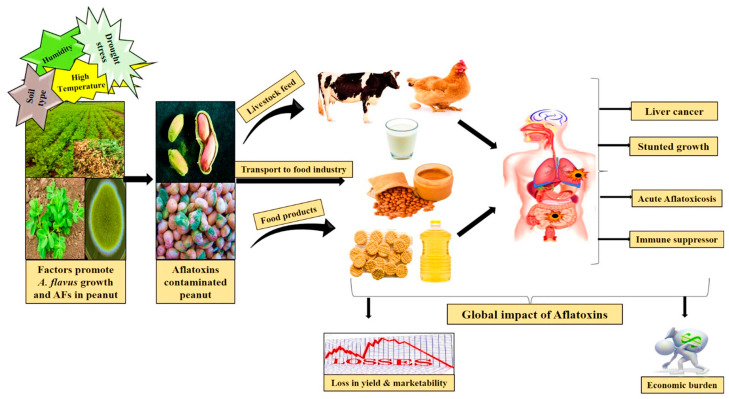
Adverse meteorological and weather conditions promote the growth of fungi, such as *A. flavus*, on food products. These fungi produce mycotoxins on the food’s surface, which can then be transmitted to humans through various channels. Mycotoxins can lead to several health issues in humans, including liver cancer, stunted growth, acute aflatoxicosis, and immune suppression. Reprint from [31]. Copyright © 2024 by the authors and ACS Publishers.

During their development on a variety of crops and food items, such as cereals, nuts, spices, and dairy products, some types of molds produce a class of naturally occurring hazardous chemical compounds known as mycotoxins [19]. Certain mycotoxins have been connected to several detrimental health effects in both people and animals (Figure 1), ranging from acute poisoning to chronic issues, including liver cancer and, in rare instances, death [20,21,22]. They are often created under favorable temperature and humidity conditions throughout the pre-harvest, harvest, and storage stages of an organism’s life cycle [21,23]. The most common mycotoxin types are generated by specific plant-pathogenic species of *Aspergillus*, *Fusarium*, and *Penicillium* and include aflatoxins, fumonisins, zearalenones, ochratoxins, and patulin [24]. It has been shown that the amount of mycotoxin contamination in agricultural products varies greatly depending on the region and is affected by the yearly weather [25,26]. Mycotoxins have been estimated to contaminate 60–80% of the world’s agricultural supply [27].

Mycotoxin contamination has a significant financial effect; the yearly worldwide estimated cost for its detection, enforcement of regulations, and mitigation efforts to control its presence in food and feed is in the billions of Euros [28]. An estimated 75 million tons of wheat in Europe—or 5% of the wheat grown for human consumption—exceeded the maximum threshold for DON contamination between 2010 and 2019. Due to this excess, the tainted wheat grain was reclassified as “animal feed”, which caused an estimated EUR 3 billion in financial losses [29]. Furthermore, [30] demonstrated that aflatoxins caused the degradation of 4.2% of wheat meant for food between 2010 and 2020, possibly resulting in an extra EUR 2.5 billion in economic losses. Therefore, it is essential to identify and control mycotoxins in crops and food items in order to maintain global consumer health, ensure food safety, and support stable economies.

### Methodology of Data Collection

Conventional detection techniques like LC-MS, HPLC, GC-MS, and ELISA provide reliable findings, but they also often produce huge, complicated datasets that need in-depth interpretation and analysis. As an alternative to conventional detection techniques, AI approaches (ML and DL) for mycotoxin detection and prediction have become more popular recently. A literature search on the impact of AI algorithms on the topic of ‘detection of mycotoxin in food’ was conducted using Google Scholar. The literature search in Google Scholar followed the same procedure as described previously [32]. Artificial intelligence-based detection of food mycotoxins, techniques in detecting fungal toxins in foods, machine learning techniques for detection of mycotoxin, deep learning techniques for detection of mycotoxin, mycotoxin detection, convolution neural network algorithm for food mycotoxin detection, artificial neural networks algorithm for food mycotoxin detection, and multi-mycotoxin detection from fungi were among the keywords used for the literature search. The literature on the subject was searched in the aforementioned database, which was published between 2014 and 2024 (up to 10 September 2024). This strategy was utilized to ensure a comprehensive coverage of potential articles on the intersection of AI (ML/DL) approaches with mycotoxin detection in food.

**Figure 2:**
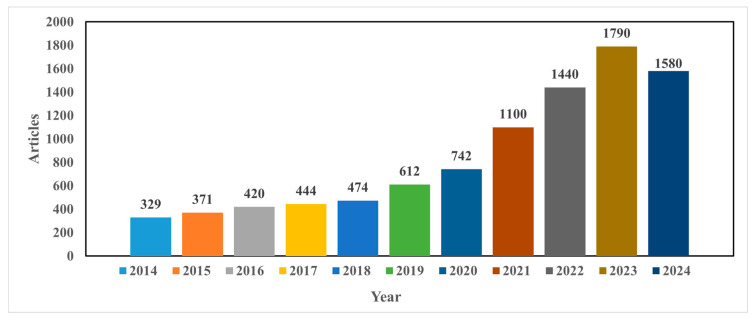
The number of published articles identified between 2014 and 2024 (until 10 September 2024) using the keyword “detection of mycotoxin in food using machine learning techniques”.

**Figure 3:**
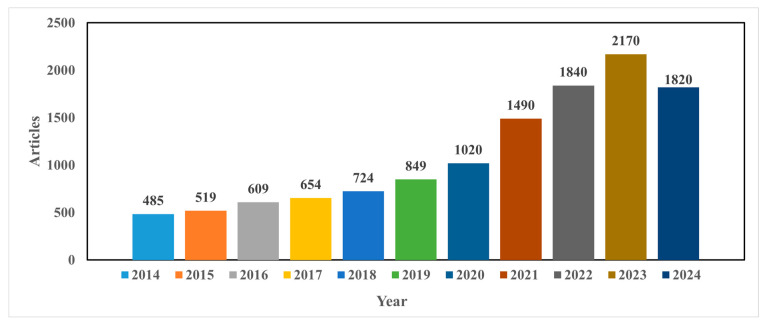
The number of published articles identified between 2014 and 2024 (until 10 September 2024) using the keyword “detection of mycotoxin in food using deep learning techniques”.

As can be seen from Figure 2, this search yielded a total of 9302 documents for “detection of mycotoxin in food using machine learning techniques”. Most articles were published in the period of 2020–2024. The search identified 742 articles published in 2020, and an increase of 1.48% was observed in 2021. Year after year, a rise in published studies was observed. Up until 10 September 2024, 1580 articles had been published, indicating that the use of ML algorithms for the detection of mycotoxin in food has been growing rapidly in recent years. Similar trends were also observed for deep learning techniques (Figure 3).

## 3. A Brief Introduction to Artificial Intelligence and Techniques

AI is a large subfield of computer science that aims to develop and improve computer systems and associated technologies that can perform activities that typically require human intellect [33,34,35]. Various cognitive skills are required to complete such activities, including reasoning, learning via experience, problem solving, language understanding, seeing, and interaction with and the changing of one’s immediate surroundings [34,36].

### 3.1. Machine Learning

ML is a branch of AI concerned with developing and improving statistical models and algorithms that can learn from past data and use that knowledge to perform tasks (like decision making or prediction) without human intervention or task-specific programming [37]. Learning (both supervised and unsupervised), as well as reinforcement learning, is a cornerstone of ML [38,39].

By assigning a target label to each input datum, supervised learning allows models to be trained with labeled data. Several algorithms form the basis of supervised learning, such as logistic regression, RF, gradient-boosted regression/classification, linear regression (LR), SVM, etc. [40,41]. When trained without labeled examples or explicit guidance, models can discover data patterns [42]. Clustering algorithms, dimensionality reduction plans, anomaly detection algorithms, association rule learning algorithms, etc., are all part of the unsupervised learning toolbox [43,44]. When it comes to machine learning, reinforcement learning means that models learn by interacting with their surroundings and receiving feedback [45].

**Figure 4:**
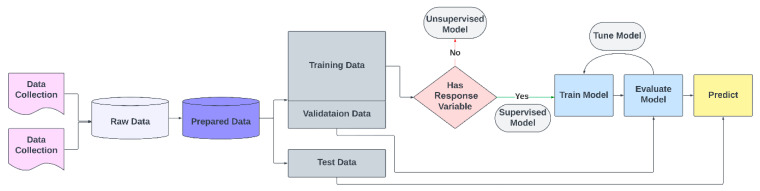
Block diagram representing the machine learning process. Reprint from [18]. Copyright © 2024 by the authors and MDPI Publishers.

The five distinct steps that ML models process are data collection, data preparation, data splitting, model selection and training, evaluation, and prediction (Figure 4). Validation and test data are two essential parts of machine learning. Validation data represent a distinct subset of the original data that is utilized after the model has been trained and validated; such data are not used during the model’s training process. In addition to helping to avoid overfitting and guarantee that the model learns to generalize from the data and produces correct predictions on fresh, unseen data, validation data also help in choosing the optimal version of the model by offering feedback on its performance. Mean square error (MSE), accuracy, sensitivity, specificity, and the Area Under the Receiver Operating Characteristic Curve (AUC) are typical performance measures for machine learning models.

### 3.2. Deep Learning

The patterns of information processing in the human brain serve as an inspiration for DL, which utilizes massive amounts of data to translate user input to labels rather than relying on pre-existing rules created by humans. DL is built using a plethora of artificial neural networks (ANNs), each offering a unique perspective on the input data. DL improves upon earlier iterations of neural networks by a significant margin. In addition, DL constructs multi-layer learning models by using transformations and graph technologies in tandem. Natural language processing (NLP), visual data processing, audio and voice processing, and other applications have all shown remarkable improvements in performance using the latest DL approaches [46,47,48,49]. Among the most well-known and widely used DL networks are convolutional neural network (CNNs) [50,51]. Nowadays, DL is all the rage, thanks to CNNs. The most important reason CNNs are so popular is because, unlike their predecessors, they can discover important traits automatically without human intervention.

DL is frequently called universal learning, as it works in almost all application fields. DL approaches do not need features to be carefully developed. Rather, the optimum traits are automatically learned in relation to the job at hand. However, resilience to typical variations in the input data is achieved. The same DL methodology may be used for various data types or applications; this method is sometimes called transfer learning (TL), which is discussed in the next section. It is also a helpful strategy when there are insufficient data. Algorithms for DL are very scalable. Applications of ResNet [37] at the supercomputing scale are common.

## 4. Artificial Intelligence Techniques for Mycotoxin Detection in Food

AI techniques offer numerous advantages, including reliability, cost-effectiveness, and the ability to handle uncertainty [52]. AI techniques can also potentially reduce processing time in several applications [53]. However, the effectiveness of AI techniques can vary depending on the specific task. The application of AI often relies on access to large, high-quality datasets. This review focuses on AI techniques for the detection of mycotoxigenic fungi and mycotoxins in foods. All nations in the world cultivate a lot of peanuts, which are both an oil crop and an import cash crop. Nutrients like fat and protein are abundant in peanuts. At the same time, as they are circulated and stored, finished peanuts are vulnerable to mildew and mold growth. Aflatoxin is a poisonous and carcinogenic chemical often found in moldy peanuts, among other food products. The olfactory visualization approach was used to identify aflatoxin B1 (AFB1) in peanuts [54]. The color component of the pre-processed sensor feature picture was optimized using an artificial intelligence approach involving a genetic algorithm (GA) with a backpropagation neural network (BPNN) as the regressor. The optimal mix of distinctive color components was used to develop a support vector regression (SVR) quantitative analytic model for the determination of AFB1 in peanuts. Two optimization techniques for SVR parameters were applied, namely grid search (GS) and sparrow search (SS). The results showed that the SS-SVR model achieved the best prediction effect, with 0.91 correlation coefficients of prediction. Other machine learning algorithms, including SVM, an artificial neural network (ANN), and an adaptive neuro-fuzzy inference system (ANFIS ) were used to efficiently detect aflatoxin and aflatoxigenic fungi in peanut seeds [55]. Images of peanut seeds were captured utilizing several illumination sources (LED, UV, and fluorescent lamps) against two backdrops (black and white) at 0, 48, and 72 h post inoculation. The research findings indicate that the use of LED light and a white backdrop, along with an artificial neural network (ANN), achieved a 99.7% accuracy rate in identifying fungal development on peanuts after 72 h. In another study, four ML algorithms (SVM, RF, multi-layer perceptron (MLP), and linear discriminant analysis (LDA)) were used to detect fungi (*A. flavus, A. niger, Penicillium* sp., and *Rhizopus* sp.) on peanut seeds [56]. Multispectral images were used to classify the fungi, and machine learning algorithms achieved exceptional accuracy in the autonomous identification of seed health conditions (90 to 100%). An SVM and deep convolutional neural network (DCNN) were employed to detect aflatoxin on peanuts using optical coherence tomographic images, demonstrating that the suggested methods could accurately identify peanuts affected with mold, with about 85% and 96% accuracy, respectively [57]. The latest AI technique, namely the transformer algorithm, has also efficiently identified mycotoxins in peanuts [58].

**Figure 5:**
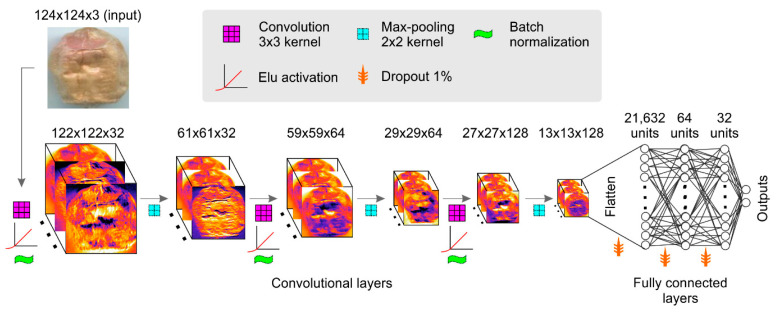
Hyperparameter tuning is first conducted using a random search method, followed by the development of a custom network architecture. To illustrate how the input is transformed to extract features, the convolutional layers are shown with their intermediate activations, representing the outputs of the convolutional network. The dimensions of each step are indicated by the numbers displayed above the layers. Reprinted from [59]. Copyright © 2024 by the authors and Agriculture, MDPI Publishers.

Wheat is an important crop for world food security. However, this crop is under attack from a variety of biotic and abiotic stressors, resulting in considerable reductions in yield and quality. Fusarium head blight (FHB), which is mostly caused by *Fusarium graminearum* Schwabe, is one of the most common and devastating fungal diseases in wheat. Using a DL technique, a CNN efficiently predicted wheat infection using ordinary RGB camera images [59]. Using a low-complexity design architecture with hyperparameter optimization, the models attained a precision of 97% in identifying FHB in seeds and 99% accuracy (Figure 5). Another research study demonstrated the effectiveness of a deep convolutional neural network (DCNN) with transfer learning in evaluating FHB on wheat [60]. The model achieved an average accuracy of 0.92 on the testing dataset using colored pictures (captured by a Canon EOS Rebel T6i, New York, NY, USA). The CNN structure was also proven effective in detecting AFB1 in wheat [61]. Data were collected using a microwave detection device to test the efficiency of the CNN model. Qualitative analysis showed that the fusion CNN model achieved perfect scores of 100% for accuracy, precision, recall, and F1 score in making predictions. Wheat was tested for the presence of deoxynivalenol, zearalenone, T-2 toxin, HT-2 toxin, fumonisins, aflatoxins, and ochratoxin using the ML technique and RF [62]. Data on crop phenology and weather, as well as satellite photos, were used to conduct the research. The model performed admirably in terms of predictions, with internal and external validation accuracies of 99% and 90%, respectively. In order to find a fast, precise, and inexpensive way to identify wheat samples contaminated with deoxynivalenol, researchers wanted to see whether electronic nose (e-nose, a biosensor) may be useful [63]. A portable e-nose called the “AIR PEN 3” (Airsense Analytics GmbH, Schwerin, Germany) with ten metal-oxide sensors for various categories of volatile chemicals was used to collect samples. Classifying data using the “Classification and Regression Trees” (CART) machine learning technique achieved an accuracy of 83%. Several other studies have also shown the efficacy of the ML, DL, and transformer models in detecting the mycotoxins in wheat [64,65,66].

**Figure 6:**
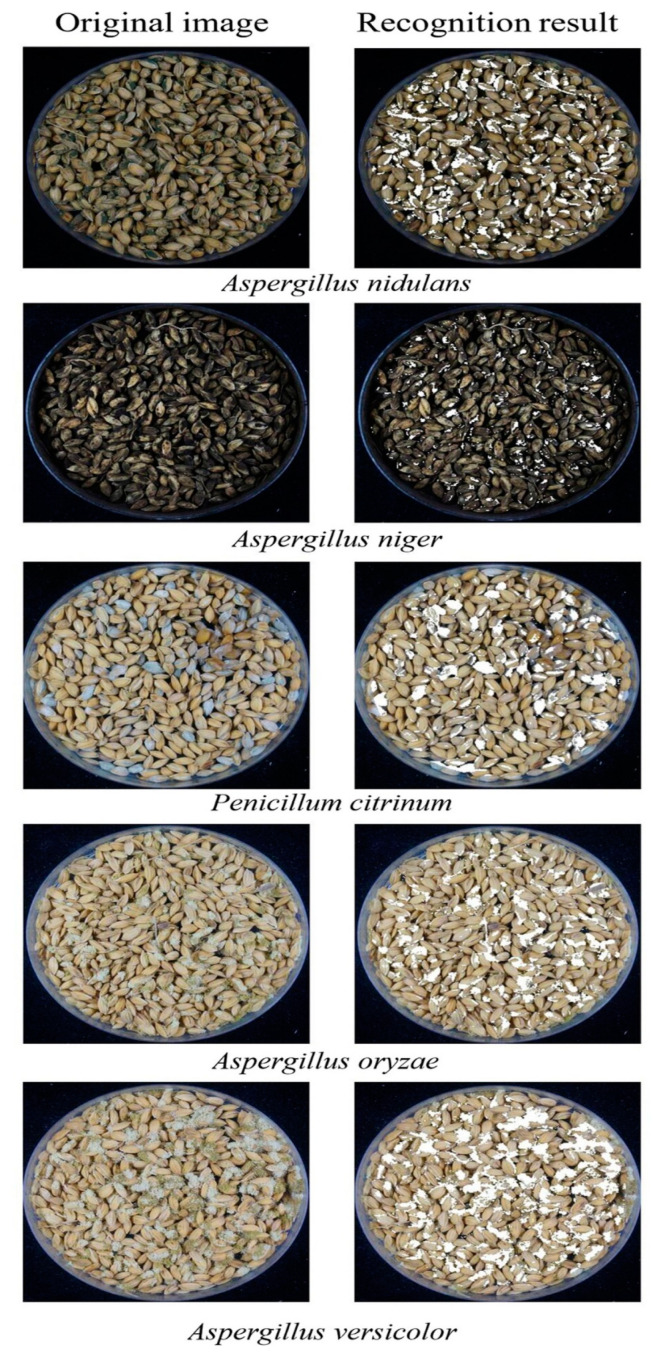
Detection of mycotoxins on wheat using a deep learning technique. Reprint from [67]. Copyright © 2024 by the authors and Scientific Report, Nature.

Particularly in Asia, rice is a staple meal that is consumed widely. After harvesting, rice is typically kept as unhulled paddy. Unhulled paddy often develops mold while being stored, losing its nutritional value for human use. *Aspergillus nidulans*, *Aspergillus niger*, *Penicillium citrinum, Aspergillus oryzae*, and *Aspergillus versicolor* mold species (Figure 6) were inoculated and cultured on samples [67]. Sample images were captured using a system made by researchers using a Sony Nex-6 camera (New York, NY, USA). SVM, BPNN, CNN, and deep belief network (DBN) models were used in the construction of mold identification techniques. The DBN model was able to identify the kind of mold in the sample photos with 100% accuracy. SVM and BPNN were also used to determine the contamination of rice using an electronic nose [68]. An old and significant cereal grain crop is barley, which is now the fourth most produced cereal in the world. In another study [69], using hyperspectral imaging and an improved CNN, the viability of categorizing deoxynivalenol levels in several genetic lines of barley kernels was assessed. Classification models were created using LR, SVM, CNN, RF, K-nearest neighbor (KNN), stochastic gradient descent (SGD), and LR, among other machine learning techniques. The findings imply that CNN has significant potential, discriminating deoxynivalenol levels of barley kernels with 89.81% accuracy.

The world’s other high-yield grain crop, maize, is also vital for many other agricultural processing sectors, including the feed, chemical, and food industries. CNN algorithms were utilized in conjunction with hyperspectral imaging (HSI) and sparse auto-encoders (SAE) to categorize the moldy maize kernel grades [70]. A CNN was used to extract image features, while SAE was employed to represent the spectral depth information. To create classification models, spectral and image information was coupled with KNN, SVM, and partial least-squares discriminant (LSD) analysis classifiers. With highly accurate recognition rates of 99.47%, the SAE-CNN-SVM model, which was built by combining SAE and a CNN with the SVM classifier, achieved the most satisfying identification result. To build a deep learning model for the monitoring of AFB1 in maize, a one-dimensional CNN (1D-CNN) and two-dimensional CNN (2D-CNN) were created [71]. The samples were collected by a near-infrared (NIR) spectrometer, and the obtained results showed that the performance of the 2D-CNN model was significantly better than that of a 1D-CNN, with a 0.9955 coefficient of predictive determination. A deep neural network (DNN) predicted AFB1 and fumonisins on maize using metrological data [72]. The primary determinant of mycotoxin-producing fungus and the subsequent contamination of maize grain is meteorological circumstances; however, the cropping technique may significantly reduce the influence of these environmental factors. The Emilia Romagna meteorological service provides hourly data on air temperature, relative humidity, and rain (in millimeters) that authors can download for research purposes upon request. With 75% accuracy, the AI model effectively predicted mycotoxins in maize. To determine the elements that substantially contribute to outbreaks of mycotoxin contamination in maize, a model was created using data on aflatoxin (AFL) and fumonisin mycotoxin contamination, as well as satellite imagery, daily weather reports, and data on dynamic geospatial soil characteristics and land use parameters [73]. Two AI algorithms were used, namely a gradient boosting machine (GBM) and a neural network (NN). NN models achieved better class-specific performance for AFL (73%) and fumonisin (85%), highlighting their accuracy for annual mycotoxin prediction. AI techniques were applied to Raman spectroscopic image data to detect aflatoxin in maize [74]. The data were classified using four different methods, namely LDA, quadratic discriminant analysis (QDA), quadratic support vector machines (QSVMs), and linear support vector machines (LSVMs). When LDA was combined with Savitzky-Golay 2nd derivative (SG2) preprocessing, the highest classification accuracy of 95.7% was attained. An RF algorithm was developed for NIR spectrometer data by applying different cut-offs to classify samples of *Fusarium*, *Fusarium*, Fumonisins, and *Penicillium* [75]. The RF model classified the toxins with 98.6% accuracy. Researchers also used the BPNN DL model to efficiently detect zearalenone, another kind of mycotoxin in maize [76].

Within the food sector, random product sampling, sensory panel descriptions of taste and fragrance, volatile and headspace analyses, and mycotoxin analyses are often used methods of quality control. Based on electronic nose analysis, classification models for *Penicillium expansum* apple spoilage and prediction models for patulin content in apples suitable for apple juice production were created [77]. Partial least-squares prediction models with strong correlations were created to predict the contents of patulin in apple juice and shredded apples. Researchers also used a voltammetric aptasensor to examine ochratoxin A, fumonisin B1, and deoxynivalenol mycotoxins and found them to be highly selective using a smartphone-integrated device [78].

Considering both monitoring cost and model performance, machine learning algorithms were applied to investigate risk-based monitoring programs for AFB1 in feed products in [79]. Historical monitoring data were utilized to determine the presence of AFB1 in feed items. To anticipate the high-risk feed batches to be taken into consideration, four different machine learning algorithms were deployed and contrasted, namely Decision Tree (DT), LR, SVM, and Extreme Gradient Boosting (XGB). With a 90% success rate, the ML algorithms identified the batches that posed the most threat. All three of the tested ML algorithms performed worse than the XGB method. A biosensor-based system was introduced for the sensitive assessment of antibiotic residues in raw cow milk. This system incorporates nanotechnology, optomechanics, and a spectral detection algorithm [80]. Four commonly used antibiotics, namely sulfadimethoxine, ampicillin, oxytetracycline, and kanamycin, were targeted by chemically connecting aptamer receptors with gold nanoparticles to create nano-biosensors. The method was found to be more sensitive and faster than the most recent state-of-the-art standardized methods for antibiotic detection. A Bayesian network also proved its efficacy in the detection of chemical hazards in milk [81].

A 1D-CNN significantly classified the multiple kinds of foods contaminated by aflatoxin [82]. Data were collected using hyperspectral equipment. With settings of epoch = 30, learning rate = 0.00005, and ’relu’ for the active function, the 1D-CNN fared the best. The maximum test accuracy for peanuts was 96.35%; for maize, it was 92.11%, and for mix data, it was 94.64%. Additionally, AI-powered ML algorithms have shown their effectiveness in creating clay-based composite materials for the removal of mycotoxins from animal feed [83]. ML models are also useful in the identification of key microbes (differentiated based on geographic region) in Mediterranean cheese [84].

CNN-based GoogLeNet, SqueezeNet, AlexNet, and ResNet50 models were used to determine aflatoxin contamination in cocoa beans [85]. Liquid mass chromatography was used to capture the imaging data, and GoogLeNet-CNN performed best with, 96.42% accuracy. Another statistical technique, namely regression analysis, was used to quantify the contamination of four classes of aflatoxins (B1, B2, G1, and G2). Data were recorded using an ultra-high-performance liquid chromatography fluorescence detector [86]. The techniques efficiently quantified mycotoxins with a high coefficient of determination (0.995). ML techniques effectively detected the contamination of coffee using NIR spectra [87].

Several studies have detected the mycotoxins using ML and DL techniques on the surface of other food products, like edible oil, almonds, figs, and alcohol [88,89,90,91,92]. Recent research has proven the efficacy of integrating Raman spectroscopy with modern data processing methods for the monitoring of edible oil quality and safety. Deep learning models such as CNNs and recurrent neural networks (RNNs) have achieved great performance in detecting AFB1 contamination in edible oils, with 100% accuracy for qualitative identification and high precision for quantitative detection [88]. Furthermore, integrating Raman spectroscopy with chemometric approaches such as partial least squares (PLS) has shown good accuracy in predicting AFB1 levels in pressing peanut oil [93]. Recent studies have looked at non-destructive approaches for the detection of aflatoxins in almonds using optical techniques. The use of fluorescence imaging in combination with deep neural networks produced excellent results, with classification accuracies ranging from 84.7 to 93.0% for contaminated samples [89]. Similarly, the combination of fluorescence spectroscopy with machine learning algorithms detected aflatoxin B in almonds with 94% accuracy [94]. Kılıç and İnner [90] developed non-invasive ways to identify aflatoxin contamination in dried figs, overcoming the constraints of manual sorting using UV light. Deep transfer learning techniques employing UV-captured pictures have shown encouraging results, with up to 98.57% accuracy. Similarly, machine learning algorithms have been used to forecast the development and mycotoxin production of Fusarium species under various conditions [92]. These investigations examined the impact of ethylene–vinyl alcohol copolymer films with essential oil components (EOCs) and antifungal drugs on fungal growth and toxin production [92,95]. The efficacy of several EOCs and antifungal treatments was assessed under a variety of temperature and water activity conditions. Random forest models outperformed other machine learning approaches in the prediction of fungal growth rates and mycotoxin production [92]. Table 1 lists the AI techniques used in the detection of mycotoxigenic fungi and mycotoxins in foods.

## 5. Challenges Associated with the AI-Based Detection of Mycotoxins in Foods

While AI helps detect mycotoxigenic food items, until technological challenges and constraints of AI systems are addressed, such techniques cannot be used to their maximum effect. Such problems include a variety of applications in the field of mycotoxin detection by AI.

1. Data quality is a must to ensure that AI algorithms have what they need for training and validation. However, obtaining large datasets covering an extensive range of mycotoxin profiles and food matrices influenced by a wide variety of environmental conditions can be challenging. For example, the rarity of annotated data for the training of AI models is a major bottleneck in transferring these methods to newly identified mycotoxins or less studied food types [72].
2. Chromatography and spectroscopy are two of the many laboratory methods used to detect mycotoxins and conduct complex chemical analyses. AI systems have to be able to decompose complex spectral or chromatographic datasets of expression patterns and integrate multiple data modalities. It remains a significant technical challenge to build AI models that can generalize well across different detection modalities and food matrices [97].
3. Overfitting is a condition whereby AI models trained on certain datasets perform well on training data but struggle to generalize to new, unknown data. Sophisticated regularization approaches and validation methodologies are needed to provide strong model generalization across various manufacturing batches, geographical locations, and environmental circumstances [62].
4. Due to the inherent complexity of the DL model, which often results in black-box predictions, it is challenging to draw conclusions based on AI-based detection. A hurdle to regulatory acceptability and confidence amongst food sector stakeholders and regulatory bodies is the interpretability of such models [98].
5. High-performance computer infrastructure and effective algorithms are among the many computational resources needed to implement AI algorithms for real-time mycotoxin detection. It may be possible to analyze larger datasets and perform real-time analyses in food processing facilities or field situations, surpassing present computing capabilities [99].
6. It would be very helpful to detect mycotoxins effectively as a result of the application of AI-based techniques in conjunction with conventional methods such as chromatography and immunoassays. Adoption in industrial processes depends on smooth integration with current procedures while using AI’s benefits in terms of speed and sensitivity [100].
7. Developing and implementing AI systems for mycotoxin detection might be expensive, as they need to be equipped with appropriate hardware, software, and knowledge. The affordability and scalability of AI technologies continue to be crucial factors in their broad adoption, especially by small-scale food producers and in areas with limited resources [18].
8. Addressing ethical issues associated with AI’s effects on food safety regulations and regulatory compliance is critical. For consumer safety and industrial adoption, AI-driven mycotoxin detection systems must demonstrate responsibility, openness, and compliance with legal requirements [101].
9. The development and implementation of AI systems for mycotoxin detection may be expensive, since they require investments in specialized hardware, software, and knowledge. When it comes to the broad use of AI technologies, the cost-effectiveness and scalability of these technologies continue to be significant factors, especially among small-scale food producers and in areas with limited resources.
10. Establishing standardized methodologies and guidelines for the detection of mycotoxins using AI is critical in guaranteeing consistency among regulatory agencies and laboratories. By harmonizing methodology and performance criteria, AI systems can be assured to meet stringent regulatory standards for global food safety testing. Researchers, industry, and regulatory authorities must collaborate to develop a consensus on validation procedures and acceptance criteria.

Researchers, industry stakeholders, and regulatory organizations must work together to overcome these technological obstacles and restrictions. More robust and dependable AI solutions for mycotoxin detection will be made possible by ongoing innovation in AI algorithms and data-gathering techniques, as well as multidisciplinary teamwork. This will eventually improve food safety and public health consequences.

## 6. Conclusions and Future Perspectives

In conclusion, the applications of artificial intelligence methods in the food industry for the detection of mycotoxin correspond to remarkable progress in food inspection and safety. The results of this study emphasize the expansion of the usage of artificial intelligence (AI) techniques, such as machine learning (ML) and deep learning (DL) for mycotoxins recognition and quantification across food categories. The number of publications in this field has grown exponentially over the last decade, demonstrating the maturation of research and an increase in interest. During the past decade, different artificial intelligence (AI) techniques, including machine learning algorithms such as support vector machines (SVMs), artificial neural networks (ANNs), and deep convolutional neural networks (DCNNs) have been able to detect mycotoxins with great accuracy and efficiency in numerous crop species, such as peanuts, wheat, maize, rice, and barley, among others. Some of these mycotoxins include aflatoxins, deoxynivalenol, and fumonisins. The use of advanced hyperspectral photography, electronic noses, and biosensors, combined with deep AI models, has dramatically improved the performance of detection techniques. Despite these advances, significant obstacles remain. Critical issues requiring continued attention include data quality and variability; the necessity of huge, annotated datasets; and the generalizability of AI models across varied settings and food matrices. Furthermore, the computational complexity and resource needs of certain AI algorithms may preclude their practical use in resource-constrained environments. The ongoing development of AI and its applications promises to improve mycotoxin detection accuracy, speed, and cost-effectiveness, resulting in improved food safety and public health consequences. As this field continues to develop, it will be essential for regulatory agencies, industry stakeholders, and academics to work together to realize the full potential of artificial intelligence and guarantee the safety and quality of food products. The potential future views that need to be addressed are discussed in the following paragraphs.

1. Future research should address these problems by creating more resilient and flexible AI models, improving data-collecting techniques, and investigating the integration of AI with new technologies.
2. Real-time AI monitoring systems can enhance food safety. AI algorithms attached to IoT sensors can be used detect mycotoxin contamination early. Machine learning algorithms can forecast contamination incidents based on past and current data, allowing for proactive food safety.
3. Future studies may improve AI systems for low-resource environments. High-mycotoxin areas may lack infrastructure. Lightweight, low-power mobile AI models or low-cost mycotoxin sensors can be developed.
4. Data augmentation may enhance labeled data quality and quantity, making it a crucial research topic. Synthetic data generation and augmentations can provide diverse and representative datasets to train more accurate and robust AI models. New semi-supervised and unsupervised learning approaches can reduce the demand for huge, labeled datasets.
5. AI algorithms can be developed to recognize and differentiate various mycotoxins in the future. Current models detect single mycotoxins, but comprehensive systems that can identify and quantify numerous may reveal contamination levels and sources.
6. Explainable AI (XAI) can be used to improve model interpretability, increasing the adoption of and trust in mycotoxin detection systems. Future research might provide decision support systems that explain model predictions and impacts. This may improve food safety management, regulatory compliance, and decision making.
7. The use of AI and blockchain technology can improve food supply chain transparency. Blockchain technology can immutably record AI system data and choices, making mycotoxin detection and management auditable. Future research might combine blockchain and AI-driven detection technologies to establish secure, transparent food safety networks.
8. Cross-disciplinary AI research might include microbiology, agronomy, and food science. AI researchers and domain specialists working together can develop new solutions to better comprehend mycotoxins, environmental conditions, and food goods.

## Figures and Tables

**Table 1:** AI techniques for the detection of mycotoxigenic fungi and mycotoxins in foods.

Mycotoxin	Food Matrix	Detection Method	AI Algorithm	Performance Measurement Parameter	Results	References
AFB1	Peanut	Olfactory visualization	GA+BPNN+ SVR+SS	Correlation coefficient	0.91	[54]
AFB1	Peanut	UV lamp, fluorescent light lamp	ANN, SVM, ANFIS	Accuracy	99.7%	[55]
*A. flavus *^a^, *A. niger *^a^, *Penicillium* sp. ^a^, *Rhizopus* sp. ^a^	Peanut	Multispectral images	SVM, MLP, LDA, RF	Accuracy	90–100%	[56]
AFB1	Peanut	Optical coherence tomography	SVM, DCNN	Accuracy	96%	[57]
Aflatoxin	Peanut	Hyperspectral imaging	Transformer	Accuracy	98.42%	[58]
*Fusarium* head blight ^b^	Wheat	RGB camera	CNN	Accuracy	99%	[59]
*Fusarium* head blight ^b^	Wheat	Canon EOS Rebel T6i camera	DCNN	Average precision	0.92	[65]
AFB1	Wheat	Microwave detection device	CNN	Accuracy	100%	[61]
deoxynivalenol, zearalenone, T-2 toxin, HT-2 toxin, fumonisins, aflatoxins, ochratoxin	Wheat	Wheat phenology data, weather data, and satellite images	RF	Accuracy	99%	[62]
*Aspergillus nidulans *^a^, *Aspergillus niger *^a^, *Penicillium citrinum *^a^, *Aspergillus oryzae *^a^, *Aspergillus versicolor *^a^	Rice	Sony Nex-6	SVM, BPNN, CNN, DBN	Accuracy	100%	[67]
*Aspergillus* spp. ^a^	Rice	Electronic nose	BPNN, SVM	Accuracy	96.4%	[68]
Deoxynivalenol	Barley	Hyperspectral imaging	CNN, LR, SGD, SVM, KNN, RF	Accuracy	89.81%	[69]
Deoxynivalenol	Wheat	Electronic nose	CART	Accuracy	83%	[96]
Aflatoxin, Deoxynivalenol	Wheat	Environment data	Transformer	Accuracy	83.33%	[64]
*Fusarium* head blight ^b^	Wheat	Hyperspectral imaging	ANN, SVM, LR	Accuracy	95.6%	[65]
*Fusarium* head blight ^b^	Wheat	Hyperspectral imaging	CNN	Accuracy	100%	[66]
Fungi ^a^	Maize	Hyperspectral imaging	SAE, CNN, SVM, KNN, LSD	Accuracy	99.47%	[70]
AFB1	Maize	NIR spectrometer	1D-CNN, 2D-CNN	Coefficient of determination	0.9955	[71]
AFB1, Fumonisins	Maize	Meteorological data	DNN	Accuracy	75%	[72]
Zearalenone	Maize	Multispectral images	BPNN	Accuracy	93.33%	[76]
Aflatoxin, Fumonisin	Corn	Weather data and satellite data	GBM, NN	Accuracy	85%	[73]
Aflatoxin	Maize	Raman spectroscopy	LSVM, LDA, QDA, QSVM,	Accuracy	95.7%	[74]
*Fusarium*-produced mycotoxin, Fumonisins, *Penicillium*-produced mycotoxin	Corn	NIR spectrometer	RF	Accuracy	98.6%	[75]
AFB1	Feed products	Historical data	SVM, LR, XGB, DT	Accuracy	90%	[79]
Aflatoxin	Peanut	Hyperspectral equipment	1D-CNN	Accuracy	96.35%	[82]
	Maize				92.11%	
	Mix data				94.64%	
Aflatoxin	Cocoa beans	Liquid mass chromatography	CNN GoogLeNet	Accuracy	96.42%	[85]
Aflatoxin	Coffee	Liquid mass chromatography	Regression	Coefficient of determination	0.995	[86]
*Aspergillus ochraceous* ^a^	Coffee	NIR Spectra	LDA, SVM, KNN, BT, NB	Accuracy	97.5%	[87]
AFB1	Edible oil	Raman spectrometry	CNN	Coefficient of determination	0.95	[88]
Aflatoxin	Almond	Fluorescence imaging	DNN	Accuracy	93%	[89]
Aflatoxin	Fig	Fluorescence imaging	DCNN	Accuracy	97.5%	[90]
Zearalenone	Alcohol	Chromatograms	RF, NN	Coefficient of determination	0.957	[92]

^a^ Fungal cell; ^b^ fungus-caused blight disease detection.

## Data Availability

No new data were created or analyzed in this study. Data sharing is not applicable to this article.
